# Factors Associated with the Performance and Cost-Effectiveness of Using Lymphatic Filariasis Transmission Assessment Surveys for Monitoring Soil-Transmitted Helminths: A Case Study in Kenya

**DOI:** 10.4269/ajtmh.14-0435

**Published:** 2015-02-04

**Authors:** Jennifer L. Smith, Hugh J. W. Sturrock, Liya Assefa, Birgit Nikolay, Sammy M. Njenga, Jimmy Kihara, Charles S. Mwandawiro, Simon J. Brooker

**Affiliations:** Faculty of Infectious and Tropical Diseases, London School of Hygiene and Tropical Medicine, London, United Kingdom; Global Health Group, University of California San Francisco, San Francisco, California; Eastern and Southern Africa Centre of International Parasite Control, Kenya Medical Research Institute (KEMRI), Nairobi, Kenya

## Abstract

Transmission assessment surveys (TAS) for lymphatic filariasis have been proposed as a platform to assess the impact of mass drug administration (MDA) on soil-transmitted helminths (STHs). This study used computer simulation and field data from pre- and post-MDA settings across Kenya to evaluate the performance and cost-effectiveness of the TAS design for STH assessment compared with alternative survey designs. Variations in the TAS design and different sample sizes and diagnostic methods were also evaluated. The district-level TAS design correctly classified more districts compared with standard STH designs in pre-MDA settings. Aggregating districts into larger evaluation units in a TAS design decreased performance, whereas age group sampled and sample size had minimal impact. The low diagnostic sensitivity of Kato-Katz and mini-FLOTAC methods was found to increase misclassification. We recommend using a district-level TAS among children 8–10 years of age to assess STH but suggest that key consideration is given to evaluation unit size.

## Introduction

Globally, the three main species of soil-transmitted helminths (STHs), *Ascaris lumbricoides*, *Trichuris trichiura*, and hookworm, are responsible for an estimated loss of 5.18 million disability-adjusted life years.^1^ The main goal of STH control programs, which typically involves mass drug administration (MDA) delivered through schools,^2^ is to reduce the prevalence of moderate or heavy intensity infections of any STH to < 1% of the at-risk population, thus eliminating infection as a public health problem.^3^ Consequently, strategies for monitoring and evaluation (M&E) and for surveillance of STH seek to provide epidemiological data on the intensity and prevalence of infection over different stages of control to inform decision-making about treatment frequency and duration (Supplemental Table 1). Geographical overlap and programmatic synergies between STH and other neglected tropical diseases (NTDs) may offer opportunities to incorporate disease-specific strategies into an integrated M&E and surveillance platform.^4^

Current guidelines for lymphatic filariasis (LF) elimination programs recommend that transmission assessment surveys (TAS) are conducted in areas, which have achieved five rounds of community-based deworming with albendazole exceeding 65% coverage.^5^ These surveys seek to 1) initially determine whether transmission has been reduced to a level so that deworming activities through communities or schools can be discontinued, and 3 years after cessation of community-based deworming to 2) evaluate potential re-emergence of transmission.^5^ As albendazole is effective against both LF and STH it has recently been proposed to integrate STH surveillance into the TAS to evaluate the impact of LF programs on STH, and thus guide the transition from community-based deworming for LF control to school-based deworming for STH control.^6^ Integrated assessment of STH within a TAS has to date been piloted in Benin, Tonga,^7^ and Sri Lanka.^8^

Although the practical advantages and potential time and cost savings of integrating STH surveillance into TAS are clear,^7^ several considerations remain unclear. First, further information is required on the ability of the TAS to estimate STH prevalence and classify areas according to treatment categories compared with other recommended M&E and surveillance approaches^2^ in different settings. Second, repeated MDA may break up large-scale patterns of risk, requiring data at higher resolution (i.e., more clusters per geographical area). Third, little is known about how variations in the TAS design (in terms of age group or evaluation unit size used) or the choice of a STH diagnostic method affect STH prevalence estimates and classifications.^7^ Finally, the cost implications of integrating STH assessment into a TAS are poorly understood, both in terms of conducting the survey designs and resulting treatment decisions.

Simultaneously addressing these issues in a single field study is unfeasible because of the logistical and cost implications of obtaining “gold standard” data and implementing multiple surveys. An alternative approach is to use a computer simulation, which has successfully been used to compare alternative survey methodologies for other NTDs^9^–^12^ and vaccination coverage.^13^ In this study, we use computer simulation and detailed field data to evaluate the performance and cost-effectiveness of the TAS design for STH assessment in Kenya. Survey designs are evaluated in terms of their ability to reliably estimate prevalence and correctly classify districts according to STH treatment categories. We also investigate the impact of varying design parameters, including the age range of sampled children and size of the evaluation unit, on performance and compare the results to standard survey designs recommended by the World Health Organization (WHO). Finally, we quantify the influence of different sample sizes and diagnostic methods on the performance and cost-effectiveness of the TAS design. Taken together, this work can inform guidelines for the optimal sampling and diagnostic approach for integrated TAS and STH surveys.

## Materials and Methods

### Overview.

This study combined computer simulation and field data from Kenya to address its aims. Such an approach allows complex sampling designs, for which probability statistics cannot be estimated mathematically, to be simulated and run repeatedly to generate evidence for decision-making. In addition, gold standard data underlying sampling simulations can be generated that vary according to defined epidemiological parameters and account for observed spatial heterogeneity of infection, to evaluate the impact on the performance of survey designs.

We used Kenya as a case study because of the availability of detailed epidemiological and programmatic data and the diversity of LF and STH transmission settings in the country. Although LF only occurs on the coast of Kenya,^14^ detailed data from STH school-based surveys before and after school-based deworming were available for both coastal and western areas of the country. Therefore, we investigated the performance of TAS and alternative survey designs in both regions, using detailed monitoring and evaluation data from the national school-based deworming program.^15^ For the remainder of the work, the term MDA is used to refer specifically to mass drug administration in the context of school-based deworming.

Pre- and post-MDA individual level infection data for hookworm, *A. lumbricoides* and *T. trichiura* were used to simulate age-specific and spatially realistic prevalence data for 6,653 schools in Western, Nyanza, and Coast provinces. These “gold standard” data were estimated for three age groups (5–16 years, 6–7 years, and 8–10 years) and sampling simulations conducted based on alternative survey designs: TAS, WHO guidelines, and district-based STH survey protocols. The impact of varying the sample size and diagnostic tool within a TAS framework was then explored in terms of performance and associated costs.

### Generation of gold standard data.

**Figure 1.**
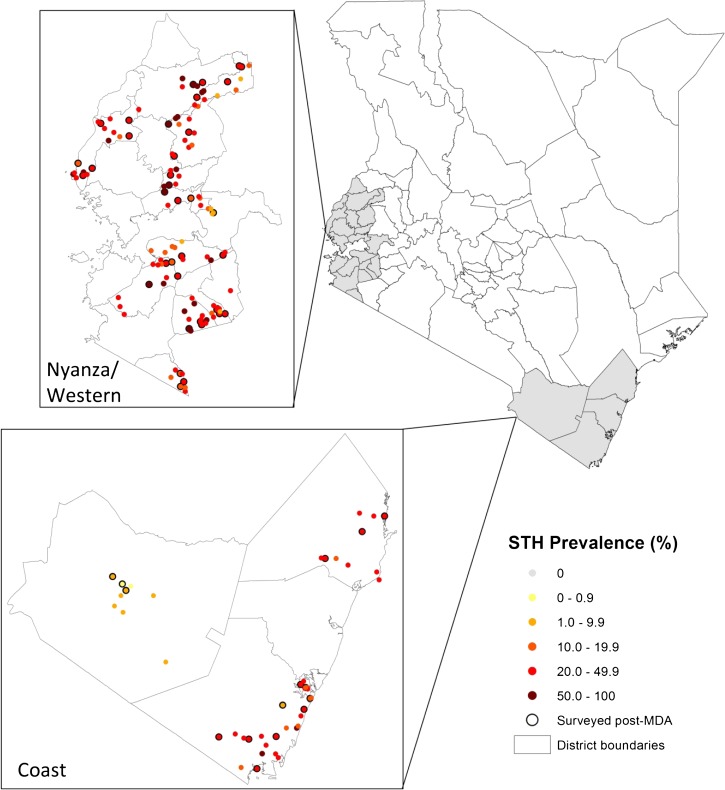
Baseline prevalence of soil-transmitted helminths (STHs) in coastal and western regions of Kenya in 2012. Schools surveyed in post-mass drug administration (MDA) follow-up are shown with a solid outline. District boundaries correspond to “old districts” from 2004 used as lymphatic filariasis (LF) implementation units.

Two empirical data sets were used to quantify age-standardized patterns in the spatial heterogeneity of the prevalence of STH species and inform generation of “gold standard” data. The first data set included individual-level baseline data from 21,528 children 3 to 21 years of age from 200 schools across coastal and western regions of the country (Figure 1).^15^ These surveys were carried out between January and April 2012, before countrywide school-based deworming, and included a random sample of 18 children from each of six classes. The second data set consisted of data from a subset of 60 schools collected 3–5 weeks after a single round of treatment with albendazole. Data from both data sets were restricted to individuals between 5 and 16 years of age, which included a median sample size of 102 in the first data set (range 63–108) and 106 (range 55–108) in the second data set.

The first step in generating age- and species-specific prevalence data at all schools in coastal and western regions involved modeling the relationship between infection status and age, using mixed effects logistic regression. As a result of the non-contiguous nature of the data, a fixed region term (coastal or western) was included to allow for differences between individuals in the two regions. School was also included as a random effect to account for correlation between individuals within schools. For each species, the fit of age included as a linear, quadratic and categorical term (5–7 years, 8–10 years, 11–13 years, and 12–16 years) was compared with Akaike information criterion values and likelihood ratio tests. Models with age and region were compared with a model only containing school-level random effect to assess model improvement by their inclusion. Having accounted for age and region, random effect values were extracted for each school for each species model and semivariograms were used to characterize spatial heterogeneity based on spatial autocorrelation structure.^16^,^17^

Prevalence data were then simulated for a larger georeferenced database of 6,653 schools, which was derived from the Kenyan Ministry of Education primary schools database.^18^ Data were simulated by first using coefficients for each species-specific model to generate age and region, school-specific log odds of infection for each species. Random effect values were then predicted for each school using a process termed conditional simulation, which uses semivariogram parameters to spatially predict random effect values for each prediction school. One thousand conditional simulations were conducted to generate 1,000 equally probable and spatially realistic “realizations” of possible random effect values at all schools.^10^,^19^,^20^ These random effect values were added to the fixed effect predictions and back transformed from log odds to generate age-specific prevalence values. This process was done for each species separately and for each of three specified age groups (6–7, 8–10, and 5–16). Prevalence of any STH species was calculated at each school for each realization assuming independent probability of infection using the following equation^21^: *pHAT* = *H + A + T - (HA) - (AT) - (HT) + (HAT)* where *pHAT* is the combined STH prevalence, *H* is the prevalence of hookworm infection, *A* the prevalence of *A. lumbricoides*, and *T* the prevalence of *T. trichiura* and the combined terms (HA, AT, HT, and HAT) are multiplicative products of the prevalence values.

To convert predicted age-specific prevalence values into numbers infected in each age group present at each school, the numbers of individuals of different age groups expected at each school was calculated. This was done by first estimating the total catchment population of each school. Population figures were estimated within theoretical school “catchment areas” using a 1 km gridded population map provided by the Worldpop project.^22^ Catchment areas were defined using Voronoi tessellation in R, which assign each 1 km grid cell to its nearest school by Euclidean distance.^23^ As population estimates were representative of all age groups, the proportion of individuals in each age group was derived from the 2011 Population and Housing Census^24^ to allow estimation of the numbers of individuals at each school in each age group. Primary school enrollment in Kenya exceeds 95%^25^; therefore, we applied a rate of 75% to generate a conservative estimate of the numbers of children in each age group likely to attend each school.

### Sampling simulations of alternative survey methods.

Simulations were used to repeatedly sample from the generated “gold standard” STH data set following the protocol of alternative survey methods, as detailed below and summarized in Table 1. In brief, two different TAS designs were considered and compared with both the WHO recommended design and the more commonly used STH design of five sites per district.

#### Transmission Assessment Surveys.

These are conducted within a geographically defined evaluation unit (EU), which either may correspond to a single LF implementation unit (IU) (usually a district) or several IUs aggregated together based on transmission risk and/or contiguous IUs with a population not to exceed 2 million. The TAS design use lot-quality assurance sampling (LQAS) to show whether the prevalence of antigenemia is above or below 2%, using the upper confidence interval (CI) associated with a true prevalence of 1% to ensure an “acceptable” level of confidence in decision-making. The precise TAS design takes into account 1) the principal vector; 2) the school enrollment rate; 3) the total size of the target population in the EU (children aged 6–7 years or in grades 1 and 2, and 4) the number of primary schools or enumeration areas.

Supplemental Figure 1 outlines the algorithm for selecting an appropriate TAS design.^5^ In brief, where the population in an EU is below a certain threshold or there are a limited number of clusters, a full census or systematic sampling of children within all schools or communities is recommended. Where the population and number of schools are above these thresholds, cluster sampling is used and the calculated (systematic) sample size is multiplied by a cluster-sample design effect of two. The number of clusters is then estimated by dividing the sample size by the average number of target-year (in this case assumed to be children aged 6–7) children per school, with a minimum of 30 clusters per EU. The LQAS analysis requires an equal-probability sample and so clusters are chosen systematically without regard to size and then selecting a fixed proportion of eligible children. Clusters are numbered by geographical proximity (subdistricts) as opposed to alphabetical order to ensure good geographic spread.

The STH sampling simulations were run according to the previous protocol, with the number of clusters calculated based on a cut-off of 2% antigenemia prevalence determined by the principle LF vector in Kenya, which is *Anopheles*.^26^ Current LF implementation units in Kenya correspond to district boundaries from 2004, and are also expected to be used for TAS EUs. As we assumed that primary school enrollment was 75%, a school-based design was used and the recommended sampling methodology and corresponding number of clusters were derived from the Manual for Survey Planners based on the calculated target population in EUs and average number of children per school.^5^ Because the population of children 6–7 years of age was ≥ 1,000 and there were ≥ 40 schools in all EUs in the study provinces, a cluster survey TAS was used. The population targeted for STH assessment in these simulations was children 8–10 years of age (third grade). The proportion of children to be assessed for STH in each school was calculated so as to achieve the currently recommended total sample size of 308 per EU for cluster sampling and 154 where systematic sampling is used.^6^ These suggested sample sizes are based on LQAS critical cut-off points, which enable classification of an EU below a given prevalence threshold based on the upper 95% probability limit, to minimize Type I error (probability of incorrectly classifying an area as below a given threshold).^6^

Existing TAS guidelines are flexible in the definition of an EU and the age range of sampled individuals. Therefore, we have also simulated alternative scenarios in which 1) contiguous districts were aggregated within the study area to form EUs with populations between 1 and 2 million, and 2) children sampled for STH were drawn from the same age group as those sampled for LF (i.e., 6–7 years of age).

#### Alternative STH survey surveys.

Current WHO guidelines assume that the prevalence of STH in school-aged children is homogenous within defined ecological zones (ecozones) and recommend a sample size of 250 school children within each zone, with 40–50 children examined in each school.^2^ Here, we evaluated the performance of a survey design based on selection of 50 children 5–16 years of age from five schools per ecozone. Information on ecozones was derived from FAO Global Ecological Zones maps (2010 update), which are based on the eco-floristic zone maps produced by Laboratoire d'Ecologie Terrestre (LET) Toulouse, France.^27^ Districts were assigned to the ecozone that represented the largest proportion of its area. In addition, because of the practical difficulties in defining ecozones, we evaluated the performance of using a standard design of 50 children 5–16 years of age from five schools randomly selected from each district.

### Analysis of performance.

The performance of each sampling strategy was quantified in terms of the proportion of times that districts were correctly classified in relation to standard prevalence thresholds used to inform MDA for STH (Supplemental Table 1): 1%, 10%, 20%, and 50%. In addition, the mean error and mean absolute error associated with survey estimates was used to quantify variability around this proportion and identify systematic bias. More detailed breakdowns of performance were generated by calculating the proportion of times districts were correctly classified within narrower prevalence bins (2.5%), across all simulations.

The previous analyses use estimates of prevalence to classify districts into MDA treatment categories based on currently recommended thresholds. It has alternatively been proposed to use decision rules to classify districts.^6^ Here, the maximum numbers positive for STH in order for an EU to be classified as below a given treatment threshold is based on the upper 95% probability limit, to minimize Type I error (probability of incorrectly classifying an area as below a given threshold) while maintaining a certain level of power in acceptance. To explore the impact of using this decision rule on survey performance and associated costs, we also explored classification into relevant treatment categories using the decision rules proposed for each threshold based on the number of positive children in the recommended cluster sample of 308 (< 10%: 18, < 20%: 44, < 50%: 66).^6^

### Impact of diagnostic method and sample size.

The impact of variation in the number of children examined and choice of diagnostic method for the performance of the TAS design was explored in an extensive analysis. First, using the district-level TAS framework, we evaluated the effect of varying the total STH sample size while sampling a constant proportion of children at each cluster surveyed for LF. Sample sizes included 100, 200, 300, and 500 individuals within each EU. Second, we evaluated the implications of using the Kato-Katz method and mini-FLOTAC method in terms of their sensitivity for diagnosing STH infection, based on species-specific estimates from a recent meta-analysis (Table 2).^28^

### Cost and cost-effectiveness analyses.

The marginal costs of adding a STH survey into different district-level TAS designs, varying the sample size and diagnostic tool, were estimated using an itemized, ingredients-based approach. Only the financial costs of the survey were considered, as the intention of this study was to illustrate the relative cost-effectiveness of alternative survey designs rather than present a full economic evaluation. Cost data associated with sampling were obtained from a cost analysis of alternative STH surveillance methods in western Kenya conducted in 2013.^29^ Unit costs are shown in Supplemental Table 2 and were divided into fixed (irrespective of number of clusters or children) and variable costs (which were dependent on the number of clusters and children). We assumed one technician was required to process STH samples per team, irrespective of the number of children sampled, as the number of children sampled per cluster never exceeded 30. We also assumed that samples would be processed on site alongside LF survey activities, each team would survey two clusters per day and four teams would be active in each EU, based on experience from previous TAS surveys. Costs associated with treatment and delivery were based on published estimates, which range from 0.15 to 0.39 US$ per delivery round per child,^30^,^31^ and applied over 4 years.

The total cost of each TAS design was calculated as sum of the survey cost plus treatment costs over all districts, for each of the 1,000 realizations. This approach allows estimation of the cost-effectiveness of each TAS design, which provides a measure of economic evaluation in which both the costs and consequences of a survey design are considered. The cost-effectiveness was calculated to be equal to the total costs for all districts divided by the number of districts receiving at least adequate treatment in each of the 1,000 realizations. This second approach does not further penalize districts that are receiving more treatment than required in the denominator.

## Results

**Figure 2:**
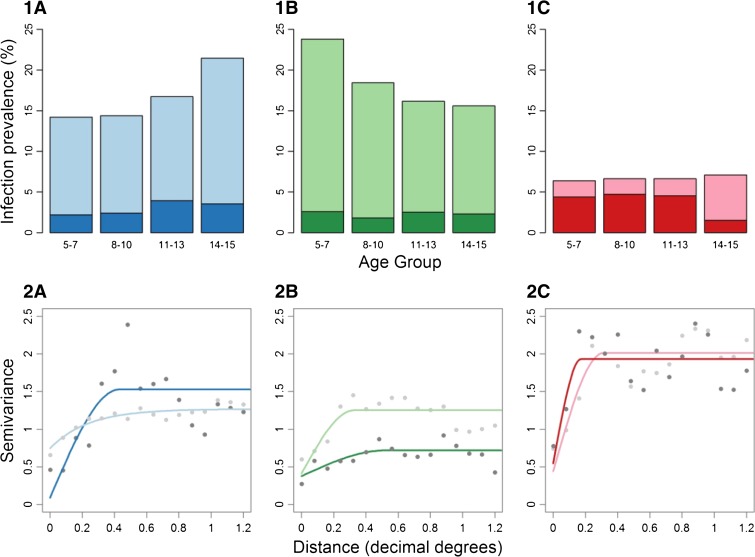
Infection prevalence: 1) by age group and semivariograms; 2) of (a) Hookworm, (b) *Ascaris lumbricoides*, and (c) *Trichuris trichiura* pre- mass drug administration (MDA) (light) and post-MDA (dark). The lower sill (point at which the semivariogram plateaus, indicating maximum value where there is still spatial structure) to nugget (minimum value at which spatial structure is present) ratio indicates less spatial structure following MDA, although these data are based on only 60 schools.

In survey data from pre-MDA settings, overall prevalence of hookworm was 15.7%, *A. lumbricoides* 18.3%, and *T. trichiura* 6.6%. There was clear heterogeneity in risk between coastal and western regions that varied by species: *A. lumbricoides* (1.0%, 23.3%), hookworm (18.5%, 14.9%), and *T. trichiura* (7.9%, 6.3%). Infection displayed contrasting relationships with age, with hookworm infection prevalence increasing exponentially with age, *A. lumbricoides* infection decreasing with age, and *T. trichiura* showing no obvious relationship (Figure 2). Age was included as a continuous variable, with models suggesting a non-linear relationship with hookworm infection, best fitted with a quadratic term, and a linear relationship between age and *A. lumbricoides*. There was no evidence of an association between age and *T. trichiura* infection (data not shown).

In post-MDA settings, overall prevalence of hookworm was 3.1%, *A. lumbricoides* 2.3%, and *T. trichiura* 4.4%. Estimates of prevalence were more similar for *A. lumbricoides* (0.1%, 3.0%), hookworm (3.2%, 3.1%), and *T. trichiura* (4.1%, 4.5%) between coastal and western regions following MDA. There was evidence that only hookworm infection prevalence varied with age and consequently, age was excluded as a covariate from other species models.

Having accounted for large-scale geographic trends and age, semivariograms of random effects revealed that in pre-MDA settings hookworm displayed spatial structure over slightly larger scales (up to 0.6 decimal degrees, 67 km) than *A. lumbricoides* and *T. trichiura,* which both showed more focal clustering up to around 0.36 decimal degrees (∼40 km) (Figure 2.2). Post-MDA data indicated clustering, with hookworm and *T. trichiura* infection becoming more focal, whereas interestingly *A. lumbricoides* became more widespread, with weaker spatial structure.

### Prevalence estimates.

**Figure 3.**
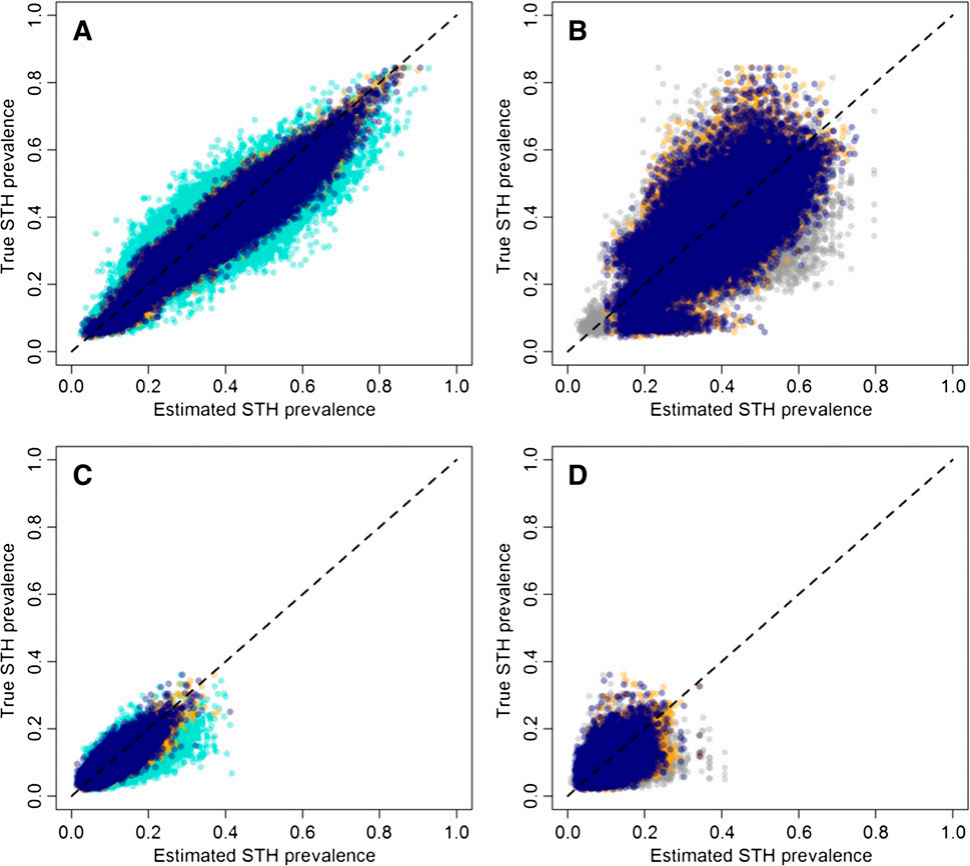
District prevalence estimates versus true prevalence estimates across all 1,000 realizations for different sampling strategies. (**A**) and (C) represent results using districts as evaluation unit (EU) in a pre-mass drug administration (MDA) (**A**) and post-MDA (**C**) setting, whereas (**B**) and (**D**) represent results using aggregated districts as EU in a pre-MDA (**B**) and post-MDA (**D**) setting. Light blue points represent a standard survey method of 50 children from five schools per district, orange points a TAS method using 8–10 year olds, dark blue points a transmission assessment survey (TAS) method using 6–7 year olds and gray points the WHO recommended approach using ecological zones as EUs. Dashed lines represent perfect correspondence.

Figure 3A and C show the prevalence estimates generated from a standard STH survey method of 50 children from five schools per district and a TAS method using districts as EU using 6–7 year olds and 8–10 year olds using pre- and post-MDA data, respectively. In both settings, the standard STH survey produced comparatively more uncertainty than the TAS designs, with larger mean absolute errors (Table 3). If EUs were assumed to be districts aggregated to form regions with up to 2 million individuals, prevalence estimates were even more uncertain, with mean absolute error rates in the 10% range (Figure 3B

and D, Table 3). Sampling 8–10 year olds tended to produce similar levels of error compared with sampling 6–7 year olds. Sampling assuming a standard design of 50 randomly selected children from five schools per district, tended to produce no bias with moderate mean absolute errors of around 3–6% in pre- and post-MDA settings. In contrast, using the recommended WHO approach, whereby districts are aggregated according to ecological zone, appeared to be the least accurate sampling strategy, with mean absolute errors of 14% and 4% in pre- and post-MDA settings, respectively (Table 3).

### Classification of districts.

In terms of the proportion of districts correctly classified, a TAS using 8–10 year olds displayed a high performance in both a pre- and post-MDA setting, classifying 88% and 76% of districts correctly, respectively (Table 3). A TAS design applied to districts aggregated with neighboring districts only classified around 72% and 57% of districts correctly in pre- and post-MDA settings, respectively, irrespective of the age group targeted. Using a standard design of 50 randomly selected children from five schools per district classified around 81% and 65% of districts correctly in pre- and post-MDA settings, respectively, although a WHO approach based on aggregating by ecozone correctly classified < 63% of districts in both settings.

Figure 4

**Figure 4.**
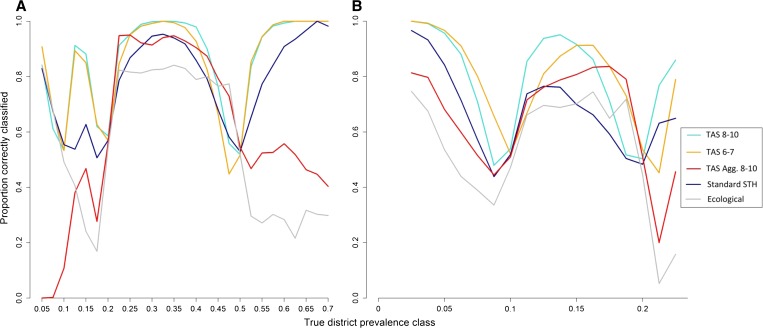
The proportion of districts correctly classified according to the true district prevalence in a pre-mass drug administration (MDA) (**A**) and post-MDA (**B**) settings. Light blue lines represent a transmission assessment survey (TAS) design using 8–10 year olds, orange represents a TAS design using 6–7 year olds and dark blue represents a standard soil-transmitted helminth (STH) design of 50 randomly selected children from five schools per district. Red lines represent an aggregated TAS design in 8–10 year olds and grey lines represent a design using five schools per ecological zone. Only categories with > 50 simulations were included to reduce random error.

presents the proportion of simulations correctly classified within each true district prevalence class, plotted against the midpoint of each 2.5% (pre-MDA) or 1.25% (post-MDA) interval. The figure highlights the inverse relationship between performance and proximity to a prevalence threshold, which reflects the mean error associated with different sampling designs. Additionally, the size of a district was observed to have an important impact on survey performance, with districts with an area greater than ∼1,500 km^2^ having higher levels of error than those with smaller areas (Figure 5

**Figure 5.**
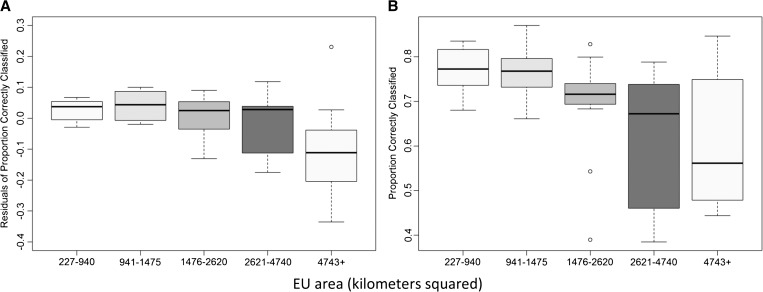
Boxplots illustrating the relationship between the area of a transmission assessment survey (TAS) evaluation unit (EU), divided into quintiles, and performance of a TAS survey design that samples 308 children aged 8–10 years, in terms of the proportion of districts correctly classified in pre- (**A**) and post-mass drug administration (MDA) (**B**) contexts. Plots include the results from district-level and aggregated EUs. As performance was strongly associated with soil-transmitted helminth (STH) prevalence in pre-MDA settings, but not post-MDA settings, boxplots in (**A)** display the residual variation in performance by EU area, after adjusting for true prevalence.

).

### Impact of sample size and diagnostic method.

Varying the total sample size within the district-level TAS design had a relatively minor impact on overall performance: although a sample size of 100 classified ∼82.8% and 75.6% of districts correctly in pre- and post-MDA settings, increasing the sample size to 500 marginally increased these proportions to 87.4 and 84.1% (Table 4). Unsurprisingly, as a result of sampling variability of a proportion, the standard deviation around prevalence estimates based on a given sample size was greatest around 0.5,^32^ but the relative uncertainty of prevalence estimates in low endemicity settings was higher at small sample sizes. As a consequence, an increase in the sample size had a greater impact in post-MDA settings where endemicity was lower and district estimates were closer to treatment thresholds.

**Figure 6.**
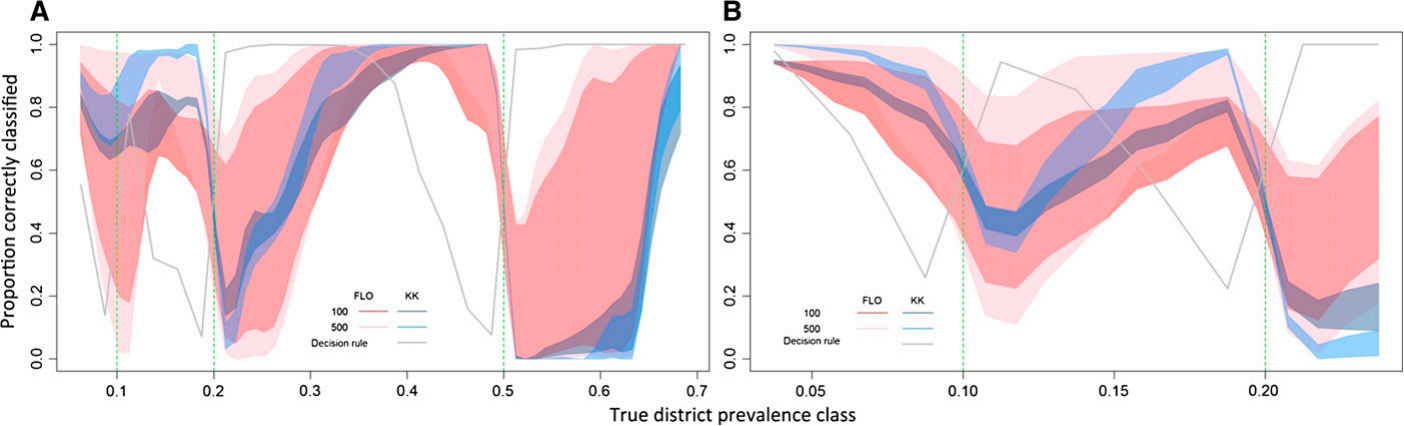
Smoothed estimates of the proportion of times each district was correctly classified in a given true prevalence class in pre-mass drug administration (MDA) (**A**) and post-MDA (**B**) settings using a district-level transmission assessment survey (TAS) design in children aged 8–10 years. Polygons correspond to the upper and lower sensitivity estimates for Kato-Katz (blue) and mini-FLOTAC (pink) using a total sample size of 100 or 500. The grey line represents the proportion of districts correctly classified using TAS decision rules, based on the midpoint sensitivity estimate for Kato-Katz and a sample size of 308. Only categories with > 50 simulations were included to reduce random error.

The sensitivity of the diagnostic method used had a greater impact on the overall survey performance compared with varying the sample size between 100 to 500 children (Table 4 and Figure 6). Compared with an assumed diagnostic method with perfect sensitivity (Table 3), the use of the Kato-Katz method and mini-FLOTAC method decreased the overall performance in pre-MDA settings by 12.5% and 6.2%, respectively. Although mid-point sensitivity estimates for *A. lumbricoides* and hookworm (the two most prevalent species) were higher for mini-FLOTAC than Kato-Katz, the greater uncertainty in mini-FLOTAC estimates limits any conclusions (Table 5 and Figure 6

).

### Survey costs and costs of misclassification.

The total marginal costs of adding an STH component to a district-level TAS framework in these areas, assuming a total sample size of 300, was estimated to be 1,249 US$ per evaluation unit using Kato-Katz and 2,318 US$ for the mini-FLOTAC method (Table 4). Although the survey costs associated with using the Kato-Katz method remained relatively constant across all sample sizes, reducing the sample size to 200 and 100 when using the mini-FLOTAC method decreased survey costs by 0.84- and 0.68-fold caused by the higher variable costs per child.

Table 5 presents the cost ratio of the total costs (survey plus treatment) per district correctly classified according to prevalence for a given sample size and diagnostic method in the TAS design, relative to that from a district-level TAS design using the Kato-Katz method and a TAS decision rule. Cost ratios in pre-MDA settings were greater than one, indicating that the costs associated with using prevalence estimates for decision-making were marginally higher than using a TAS decision rule. Increasing the sample size reduced the cost-effectiveness of using a decision rule with the standard TAS design (sample size of 308). By contrast, cost ratios were below one in post-MDA settings, suggesting that classifying districts based on prevalence estimates was more cost-effective than using the TAS decision rule (Table 5). Results additionally indicate that the higher sensitivity associated with mini-FLOTAC balanced out its higher costs, resulting in similar estimates as cost-effective as Kato-Katz (Table 5).

## Discussion

The TAS provides a logical and practical platform to integrate STH surveys with ongoing surveillance for LF. However, optimizing the protocol for STH surveillance requires consideration of a number of key components, including the effect of varying the survey platform and population (age group), sampling strategy (number of clusters and sample size), and choice of diagnostic method. This study has provided the first assessment of the impact of these operational choices on performance using computerized simulations, providing the means to compare a full range of designs in a realistic epidemiological context. Based on these results, we recommend sampling children 8–10 or 6–7 years of age using a district-level TAS to classify areas according to the prevalence of STH in coastal and western regions in Kenya. Although there is uncertainty around diagnostic sensitivity estimates of mini-FLOTAC, caused by a lack of data, our results suggest that the Kato-Katz and mini-FLOTAC methods have comparable cost-effectiveness. After accounting for diagnostic variability, the use of a TAS decision rule (based on the upper 95% CI) for STH classification increased the performance of a TAS design, as compared with classification based on prevalence estimates; however, the cost-effectiveness of this approach will diminish as the prevalence decreases or the diagnostic sensitivity increases, because of costs associated with higher Type II error.

A key finding from our analysis is that performance of the TAS design for assessing STH is largely a function of the sampling resolution and geographic heterogeneity of STH infection within a given area. Before school-based deworming (pre-MDA) in Kenya, the TAS design classified a greater proportion of districts correctly (88%) compared with the standard STH design (81%) if conducted at the district-level, because of the greater number of clusters sampled. However, when districts were aggregated into larger geographical evaluation units (up to 2 million population), the proportion of districts correctly classified using a TAS design sampling 8–10-year old children decreased to 72% in pre-MDA settings. The district-level TAS designs maintained their relatively high performance after school-based deworming (post-MDA), despite overall lower levels of error and poorer performance for all survey designs. These seemingly contrary results reflect 1) decreased variability between sites within a given evaluation unit, 2) closer proximity to a threshold caused by the overall reduction in prevalence, and 3) more focal spatial clustering after MDA (Figure 2.2). Although smaller geographic areas are likely to contain more similar prevalence levels (dependent on the range of autocorrelation), there is likely to be more heterogeneity within a geographic region that is far larger than the scale of spatial clustering. As a consequence, the proportion of variation between clusters than within clusters increases, which effectively increases the design effect required to correct for cluster sample designs. The negative impact on performance was highlighted in Figure 5, which showed that districts over 1,500 km^2^ had a lower performance than districts with a smaller geographic area. A further concern using large, aggregated geographic areas may be heterogeneity in the underlying population distribution. In Western Kenya, there is a high, relatively uniform population density and aggregated EUs cover a relatively small geographic area. Other settings with lower populations may have much larger areas aggregated together, resulting in greater variation in risk within aggregated evaluation unit and increased misclassification of underlying districts compared with a district-level design. The use of probability proportional to size (PPS) to select sites may compound this effect where the underlying population density varies substantially between districts within an aggregated evaluation unit, caused by underrepresentation of some districts. Thus, in implementing the TAS design for STH surveys, program staff should pay careful consideration to the optimal EU size.

In contrast, our results suggest that sampling a subset of children from the standard 5–16 years age range and varying the sample size between 100 and 500 within a district-level TAS framework has a minimal impact on performance or cost-effectiveness. Children 8–10 years of age may provide a marginally more representative sample than children 6–7 years of age, but the impact on survey performance was inconsequential. This finding is supported by a pilot STH assessment within a TAS conducted in Benin.^7^ Although STH prevalence estimates from third grade children were marginally higher than first and second grade children, the difference was not statistically significant. The TAS protocol used in this study varied the total sample size and sampled a fixed proportion from each selected site, as opposed to surveying only a subset of clusters for STH and maintaining a constant sample size. This approach is more robust epidemiologically, easier to implement from a logistical point of view, and is consistent with early recommendations on conducting STH assessment within a TAS.^6^ Specifically, distributing a sample of a given size so that the number of clusters approaches the number of individuals will effectively reduce correlation between individuals, providing an EU-level prevalence estimate that is more similar to a simple random sample. It should be noted that variation in the sample size may have a greater impact in a TAS design that uses aggregated EUs as explained previously, caused by the scale of clustering and heterogeneity of STH within a given area. In a post-MDA setting, the range of autocorrelation was found to be smaller, and more importantly perhaps, STH prevalence estimates were lower and closer to decision thresholds. As a consequence, the precision gained from increasing the sample size may have a greater impact on performance in this setting. Current guidelines recommend that TAS sample sizes are inflated by a design effect of two when cluster random sampling is used. As design effects are clearly scale- and context-dependent, the results from this study highlight that the design effect should consider the size of the EU as well as history of MDA.

It is well recognized that diagnostic requirements of STH,^33^ and indeed of other NTDs,^4^ are dependent on endemicity levels and programmatic aims, and that generally the sensitivity of a diagnostic must be higher in later stages of a control program to ensure higher relative precision around lower decision thresholds.^34^ Our results show that the diagnostic error introduced by using either Kato-Katz and mini-FLOTAC methods reduced the performance of the TAS design in pre-MDA settings, but that the lower sensitivity associated with these methods appeared to improve performance in post-MDA settings (Tables 3 and 4). This is likely caused by the relative position in relation to decision thresholds: in post-MDA settings, districts were more likely to be below a threshold than above it. As a result, a diagnostic with lower sensitivity is less likely to misclassify a district in a higher prevalence category. Two further points should be considered to aid interpretation of these results. First, estimates of diagnostic sensitivity for mini-FLOTAC are relatively few^28^ and have mainly been conducted in low prevalence settings. Consequently, there is a high degree of uncertainty around these estimates that are likely to improve as further studies are conducted in higher prevalence settings. However, diagnostic sensitivity for detection of any STH species in a high prevalence setting in western Kenya has been found to be comparable between Kato-Katz and mini-FLOTAC for both single and consecutive day samples.^29^ Second, the overall diagnostic performance depends on the species-specific sensitivity and, therefore, we expect this to vary with the relative levels of infection with different helminth species. Kato-Katz has a particularly low sensitivity to hookworm and thus will exhibit lower overall performance in areas where it makes up a greater proportion of infections.

Finally, the impact of using a TAS decision rule to classify districts according to prevalence thresholds was explored in this analysis. These simulations suggested that use of a threshold value based on the upper probability limit balanced the low sensitivity of the diagnostic methods and improved survey performance, compared with basing decisions on prevalence estimates. This approach effectively biases classification in favor of reducing Type I error (probability of incorrectly classifying an area as below a given threshold), at the cost of a greater risk of misclassifying districts in a higher prevalence category. This trade-off may be justified in an elimination context and where the perceived consequences of failing to treat an endemic area are high. However, the justification for this design must be balanced against the aims of the program and costs associated with treating areas where MDA is not required. In the context of STH control, these costs are likely to be very large and may not be warranted where morbidity control remains the main objective. This was particularly apparent in post-MDA settings, where endemicity was lower and there is a greater cost associated with misclassification around the 10% threshold (Table 5). Furthermore, treatment costs associated with this type of misclassification will be more problematic using more sensitive diagnostic methods, as a result of the greater risk of misclassification into higher prevalence categories. Alternative approaches might question the use of districts (or any administrative level) as IUs, given that many schools will be misclassified even when a perfect survey design is used that classifies all districts correctly. The costs associated with decision-making over such large scales are high and suggest the value of targeting on a school by school basis using predictive risk mapping.

There are a number of limitations of the methodology used in this study. First, although these simulations are realistic for this context, they are representative of a specific epidemiological setting. The endemicity levels, scale of clustering, degree of heterogeneity, geographic size of evaluation units, and population distribution will differ between countries and will impact on the overall performance of a given survey design. Second, diagnostic sensitivity is likely to vary with intensity of infection, thus we expect diagnostic performance to be relatively low in a TAS context after multiple rounds of community-based deworming in the context of LF control.^35^ These simulations used an average value for diagnostic sensitivity, whereas more realistically we would expect the diagnostic performance to be lower at lower prevalence values and higher in high prevalence settings. The lack of data on diagnostic performance in different settings limits our ability to adequately model this variation, although it could theoretically be incorporated into these types of simulation models. Finally, there are likely to be important logistic implications of diagnostics and survey designs that are beyond the scope of this study and best explored in the field to develop operational protocols. Although this study supports the epidemiological basis for sampling a subset of children within the age range of 5–16 years, the logistics of sampling the same or different children within a site may be important. Furthermore, although the number of children sampled per cluster must be adequate to provide robust treatment decisions, there are likely to be important trade-offs balancing the size of a team, the number of schools that can be covered in a day, transportation time, processing times for samples and costs associated with the above considerations.

The key findings from this study support the use of TAS as a platform for STH surveillance, and more specifically a district-level TAS sampling children 8–10 or 6–7 years of age. Furthermore, this work identifies the sampling resolution of the TAS design as a critical factor influencing its performance and suggests a need for further work to quantify the relationship between the spatial resolution of surveillance data in different epidemiological settings and the performance of survey designs. Computerized simulations provide a cost-effective tool for exploring these issues and for evaluating different sampling strategies before evaluation in the field, particularly as disease-specific initiatives are scaled up and further opportunities to integrate STH monitoring and evaluation are identified. Realistic estimation of the impact of diagnostic performance and variation in underlying spatial patterns of infection on survey performance will be aided by collection of data in the field in different epidemiological contexts. Collection of empirical data on a number of model parameters will allow more widespread and realistic use of simulations as a platform for surveillance assessment. For example, detailed species-specific estimates of diagnostic performance and parasite aggregation parameters in different endemicity settings could allow diagnostic sensitivity to be modeled in relation to intensity of infection. Furthermore, disaggregated STH prevalence data may be used to define spatial autocorrelation and heterogeneity in different contexts and allow simulation of more realistic gold standard data. We propose an iterative process, in which sampling scenarios are initially parameterized using existing data to evaluate alternative designs and understand the influence of different factors, before field evaluation and detailed epidemiological data collection to refine simulations.

## Supplementary Material

Supplemental Datas.

## Figures and Tables

**Table 1:** Overview of survey design characteristics and initial values for transmission assessment survey (TAS) and current recommended approaches for soil-transmitted helminth surveillance

Design	Evaluation unit (EU)	Number clusters	Total sample per EU	Age range (years)	Diagnostic
TAS	District	≥ 30*	308	6–7/8–10	Gold standard
	Aggregated districts	≥ 30*	308	6–7/8–10	Gold standard
WHO	Ecological zones	5	250	5–16	Gold standard
Standard	Districts	5	250	5—16	Gold standard

* Number of clusters determined according to TAS design based on population size and vector (*Anopheles*).

**Table 2:** Components used in the standard TAS evaluation and parameters varied to assess the impact of sample size and diagnostic sensitivity

Component	Standard TAS design	Varied parameters		
Evaluation unit	Districts	–		
Age range	8–10 years	–		
Sample size	Number clusters: 30+	Number clusters: 30+		
	Total sample: 308	Total sample: 100, 200, 308, 500		
Diagnostic*	Gold standard	Kato-Katz	*A. lumbricoides*	63.8 (59.1–68.6)
			*T. trichiuria*	82.2 (80.1–84.5)
			Hookworm	59.5 (56.9–62.2)
		mini-FLOTAC	*A. lumbricoides*	75.5 (54.0–95.9)
			*T. trichiuria*	76.2 (33.9–99.4)
			Hookworm	79.2 (72.7–85.9)

* Lower, midpoint, and upper sensitivity estimates correspond to those reported in Table 3 and derived from Nikolay and others (in press).^31^.

TAS = transmission assessment survey.

**Table 3:** Performance of TAS, district-based and ecozone-based STH survey designs in coastal and western regions of Kenya described in Table 2. Simulations assume a perfect diagnostic sensitivity and specificity

Survey method	Pre MDA			Post MDA		
	Mean error*	Mean absolute error†	Proportion districts correctly classified‡	Mean error*	Mean absolute error†	Proportion districts correctly classified§
TAS, 6–7 y	0.02	0.04	0.86	0.01	0.02	0.77
TAS, 8–10 y	0.02	0.03	0.88	0.01	0.02	0.76
TAS, aggregate, 6–7 y	−0.02	0.10	0.71	0.01	0.03	0.64
TAS, aggregate, 8–10 y	−0.03	0.11	0.72	0.02	0.03	0.62
STH survey (districts)	0.02	0.06	0.81	0.02	0.03	0.65
STH survey (ecozones)	−0.00	0.14	0.63	0.03	0.04	0.52

* Mean of the estimated prevalence minus the true prevalence.

† Mean of the absolute value of the estimate prevalence minus the true prevalence.

§ Proportion of times districts were correctly classified in relation to the MDA prevalence thresholds defined in Table 1.

TAS = transmission assessment survey; STH = soil-transmitted helminth; MDA = mass drug administration.

**Table 4:** Impact of variation in sample size and diagnostic sensitivity of Kato-Katz and mini-FLOTAC on performance and cost*

	Proportion correctly classified†					
	Pre-MDA		Post-MDA		Survey costs	
Sample Size	Kato-Katz	mini-FLOTAC	Kato-Katz	mini-FLOTAC	Kato-Katz	mini-FLOTAC
100	72.1 (69.2, 74.7)	80.0 (66.2, 82.1)	71.8 (71.1, 72.1)	73.2 (70.0, 71.1)	1144	1589
200	74.4 (71.7, 76.8)	81.5 (68.0, 84.8)	77.0 (75.5, 77.2)	79.5 (72.6, 76.7)	1191	1948
308	75.5 (72.3, 77.5)	81.8 (68.8, 85.8)	78.4 (77.3, 79.4)	81.2 (72.9, 78.6)	1249	2318
500	76.1 (73.8, 77.7)	82.2 (69.4, 86.9)	80.0 (78.6, 81.2)	83.7 (72.9, 81.3)	1344	2868

* Performance is measured by the proportion of times that districts were correctly classified in relation to standard prevalence thresholds by alternative district-level TAS designs in pre- and post-MDA contexts.

† Corresponding to midpoint, lower and upper estimates of diagnostic sensitivity.

MDA = mass drug administration.

**Table 5:** Median and 95% confidence interval of the ratio of total cost per district receiving adequate treatment (or better) based on prevalence estimates using Kato-Katz and mini-FLOTAC in pre-MDA and post-MDA contexts, compared with the median total cost per district receiving adequate treatment (or better) using TAS decision rules and Kato-Katz*†

Sample size	Pre-MDA				Post-MDA			
	Cost ratio (per adequate treatment)				Cost ratio (per adequate treatment)			
	Kato-Katz		Mini-FLOTAC		Kato-Katz		Mini-FLOTAC	
100	1.14	1.01, 1.36	1.08	0.92, 1.44	0.88	0.73, 1.04	0.88	0.76, 1.03
200	1.11	0.99, 1.31	1.05	0.91, 1.40	0.85	0.72, 1.02	0.85	0.73, 1.02
308	1.10	0.99, 1.30	1.04	0.89, 1.38	0.84	0.70, 1.00	0.84	0.72, 1.01
500	1.09	0.99, 1.28	1.03	0.89, 1.36	0.84	0.70, 0.99	0.83	0.71, 1.01

* Estimates include survey and treatment costs and incorporate diagnostic and sampling uncertainty by modeling the upper and lower 95% confidence interval associated with the low, high and midpoint estimate of diagnostic sensitivity. When the costs for a given prevalence design are equal to the costs of a standard TAS design using a decision rule, then the cost ratio will be one. A cost ratio greater than or less than one indicates respectively higher or lower costs per district correctly classified compared with the standard TAS with decision rule.

† Corresponding to midpoint, upper and lower estimates of diagnostic sensitivity.

MDA = mass drug administration; TAS = transmission assessment survey.
