# Supplementary material for: Factors Associated with the Performance and Cost-Effectiveness of Using Lymphatic Filariasis Transmission Assessment Surveys for Monitoring Soil-Transmitted Helminths: A Case Study in Kenya
Source: Am J Trop Med Hyg. 2015 Feb 4;92(2):342–53. doi: 10.4269/ajtmh.14-0435 (PMC4347340; doi:10.4269/ajtmh.14-0435)
Supplement: Supplementary file 1 [file SD4.pdf]

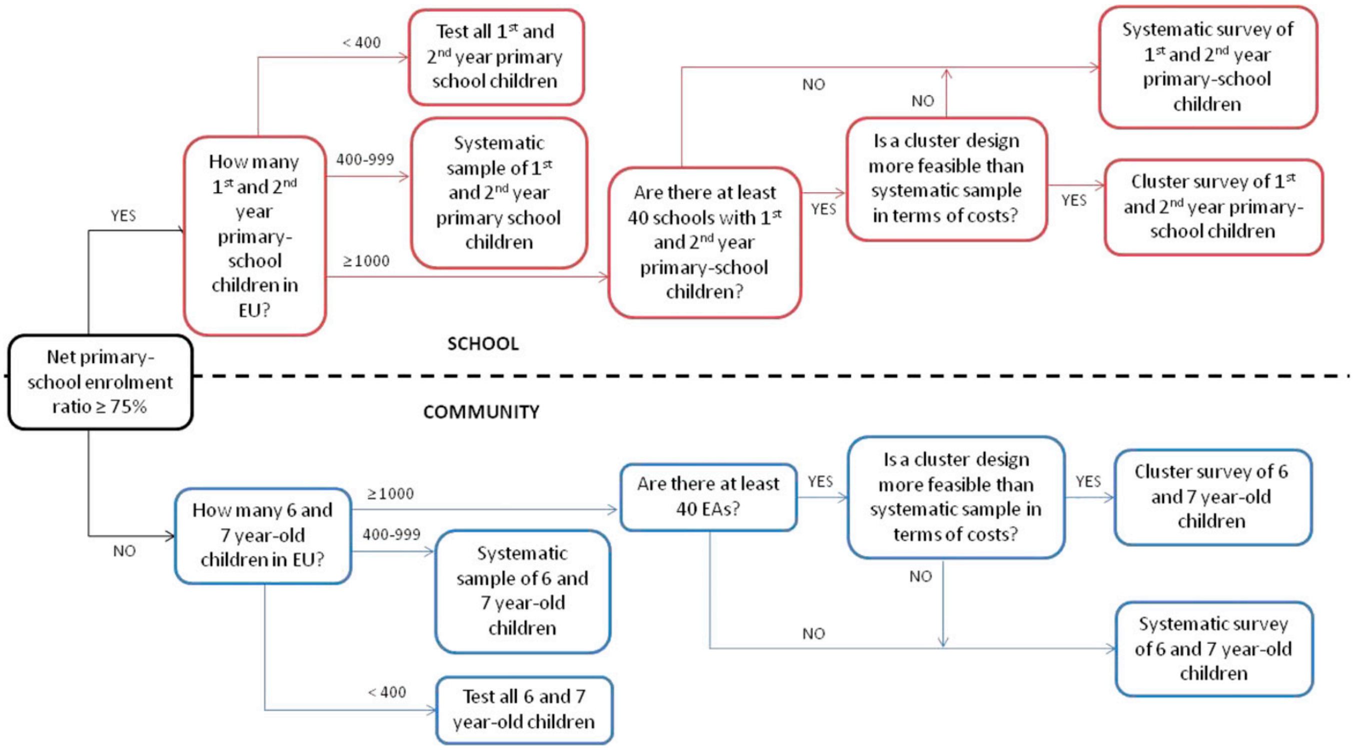

Supplemental Figure 1. Decision algorithm for choice of transmission assessment survey (TAS) design in areas where *Anopholes* or *Culex* is the principal vector. Adapted from WHO (2011).[28]

Supplemental Table 1

Current guidelines for mass drug administration (MDA) against soil-transmitted helminths (STHs)

STH prevalence category	Recommended treatment at baseline	Recommended treatment after 5–6 years of MDA
< 1%	No MDA necessary	No MDA necessary
1–10%	No MDA necessary	Maintain MDA once every two years
10–20%	No MDA necessary	Maintain MDA once per year
20–50%	MDA once per year	Transmission still intense; maintain existing MDA frequency
≥ 50%	MDA twice per year	This is a sign that the strategy was unsuccessful at controlling STH; increase frequency of MDA to three times per year

SUPPLEMENTAL TABLE 2

Itemized costs for STH assessment in Kenya using Kato-Katz or Mini-FLOTAC, used to inform the district-level TAS sensitivity analysis*†

Cost type	Item	Unit cost ($)	Units required
Materials (fixed)			
Shared	Microscope	0.47‡	4
	Microscope counter	17.83	4
	Markers	1.43	4
	Pens	0.14	8
	Pencil	0.12	8
	Erasers	0.48	4
	Pencil sharpener	0.06	4
	Buckets	1.78	4
	Tape	0.19	4
Kato-Katz	Forceps	2.38	4
	Cellophane (roll)	50.00	1
	Glycerine (bottle)	17.83	1
	Malachite green (bottle)	11.88	1
Mini-FLOTAC	Hydrometer	6.42	4
	Timer	11.88	4
	50 mL measuring cylinder	3.15	4
	2L beaker	6.06	4
Personnel (fixed)			
	Laboratory technician	47.54	4
	Cleaner	8.32	1
Consumables (variable by children)			
Shared	Toilet paper	0.26	5–25
	Disinfectant	4.75	1–2
	Liquid detergent	4.99	1–2
	Toothbrush	1.07	2–4
Kato-Katz	Frosted slides	7.13	2–10
	Stool sieves/template	0.21	100–500
	Stool polypots	0.11	100–500
Mini-FLOTAC	Saturated NaCl (500g)	4.75	2–10
	Reading disk	8.03	40–145
	Fill-flotac	3.21	40–145
	Squeeze pipets (box of 100)	0.18	4–8
Consumables (variable by site)§			
	Labels	0.31	4
	Latex gloves	11.29	4
	Wooden spatula (box of 50)	3.80	4
	Dettol hand soap (bottle)	2.38	4
	Bin bags	0.59	16
	Paper towels	2.91	8
	Newspaper	0.28	4

*Costs are based on the assumption that four teams are used per district and only one laboratory technician will be required per team and a single cleaner per district, based on the limited number of children examined per site.

†The number of units required for one district are shown with ranges according to 100–500 children per district.

‡The microscope cost calculation used an annuitization factor to account the microscope lifespan, providing an estimate of the cost of usage per day.

§These would be expected to vary when there are larger differences in site number. However, with 30–33 sites, no variation in the quantity of these items.

STH = soil-transmitted helminth; TAS = transmission assessment survey.
